# How environmental movement constraints shape the neural code for space

**DOI:** 10.1007/s10339-021-01045-2

**Published:** 2021-08-05

**Authors:** Kate J. Jeffery

**Affiliations:** University College London, London, UK

**Keywords:** Spatial memory, Neural encoding, Place cells, Grid cells, Navigation, Affordance

## Abstract

Study of the neural code for space in rodents has many insights to offer for how mammals, including humans, construct a mental representation of space. This code is centered on the hippocampal place cells, which are active in particular places in the environment. Place cells are informed by numerous other spatial cell types including grid cells, which provide a signal for distance and direction and are thought to help anchor the place cell signal. These neurons combine self-motion and environmental information to create and update their map-like representation. Study of their activity patterns in complex environments of varying structure has revealed that this "cognitive map" of space is not a fixed and rigid entity that permeates space, but rather is variably affected by the movement constraints of the environment. These findings are pointing toward a more flexible spatial code in which the map is adapted to the movement possibilities of the space. An as-yet-unanswered question is whether these different forms of representation have functional consequences, as suggested by an enactivist view of spatial cognition.

## Introduction

Spatial cognition has been an intensive focus of study in psychology for many decades, and in neuroscience ever since the 1970s when O’Keefe reported the discovery of “place cells” in the rat hippocampus (O’Keefe and Dostrovsky 1971), opening the door to the neuroscientific understanding of the representation of space. Place cells are now considered to form the core of a memory system that is built upon the foundations of a spatial map, and the question of interest here is how this map, often called the cognitive map, is structured. Answering this question will shed light on how space is mentally constructed and how this representation is used: information that could be useful in designing built spaces. Here, evidence will be presented suggesting that the cognitive map is not fixed and rigid, like an artificial map, but rather is highly situation-dependent and malleable. This implies that—like other types of perception—its function is not to report on objective reality but rather to create a subjective percept: most likely one that is tailored to the needs of a given situation.

**Fig. 1:**
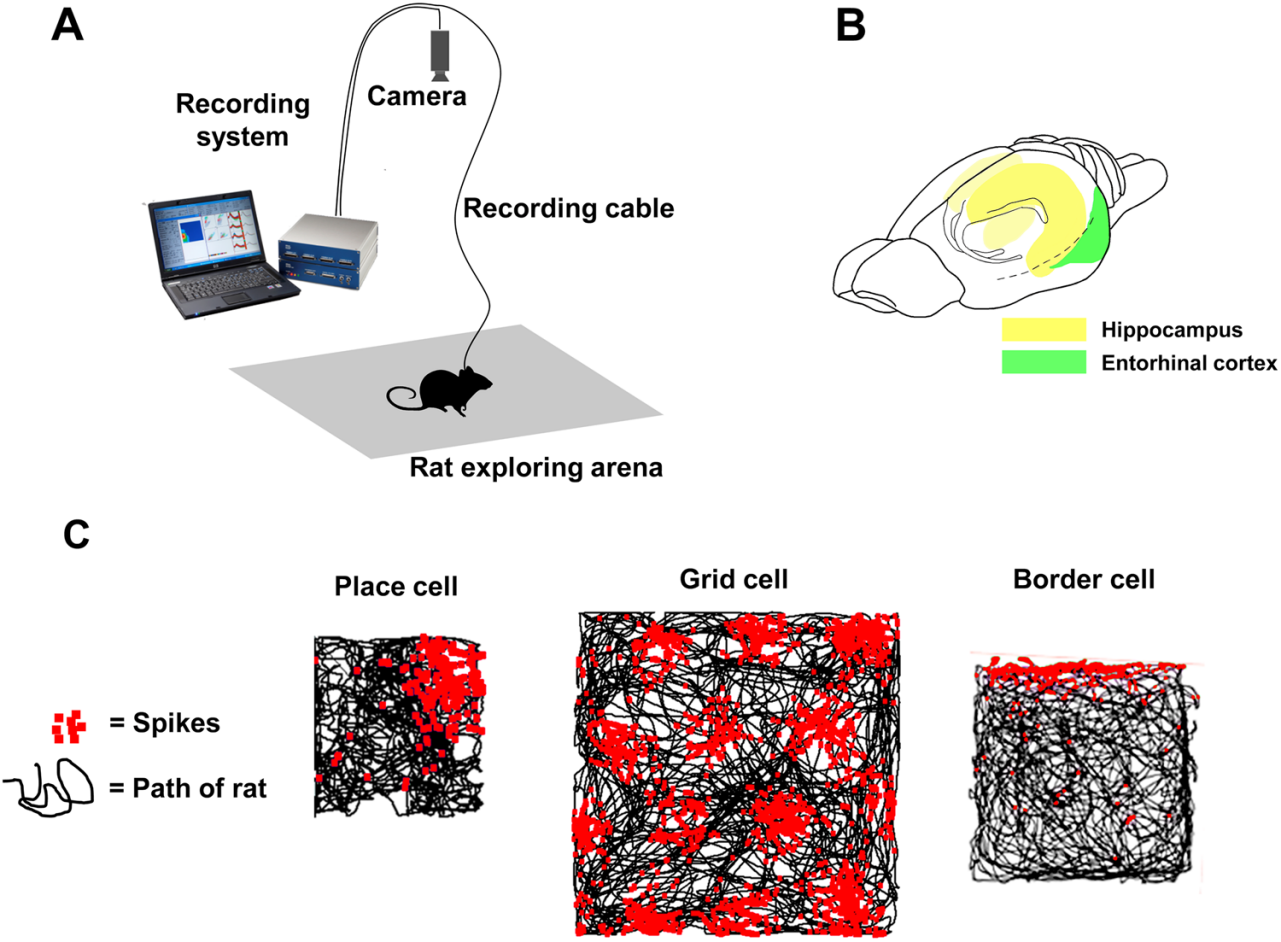
Recording of spatial neurons in the rodent brain. **A** Schematic of the experimental setup. **B** Diagram of the rat brain showing the regions containing place cells (hippocampus) and grid and border cells (entorhinal cortex). **C** Examples of the firing patterns from a place cell, grid cell and border cell. Each “spike” is an action potential emitted by a single neuron, and the pattern of spikes that builds up as the animal explores the arena congregate in characteristic areas, called firing fields. Note that for the grid cell, there are multiple firing fields and these are regularly distributed across the environment, revealing the integration of directional information (which orients the rows of fields) and distance information (which spaces them apart evenly). Source: Figshare https://doi.org/10.6084/m9.figshare.14906646 licensed under CC-BY license

Place cells are found when animals explore a space while signals are recorded from neurons in the hippocampal region. They are so-called because of their propensity to be active (fire) only when the animal enters certain regions of the environment (Fig. 1) called place fields or firing fields. The properties of place cells were thoroughly characterized early on and they were shown to be multi-modal, non-topographic (that is, not spatially arranged in the brain in a way that maps to the outside world), sensitive to environmental change and present in all mammals investigated. Their discovery immediately led to a question: how does a place cell know when it should fire? How does it know where the animal is?

Many recordings of neurons in brain regions sending inputs to the hippocampus have investigated what information they bring to place cells (reviewed in Grieves and Jeffery 2017). Most surprising and interesting of all have been the grid cells (Rowland et al. 2016). Found in the neighboring entorhinal cortex, these cells fire, like place cells, in localized regions, but unlike place cells these regions are not oval or irregular but are approximately circular. More surprisingly still, there are multiple regions per cell and these are evenly spaced, arranged in neat rows that together form a grid-like pattern (hence the name) long known to mathematicians as “hexagonal close-packed” (Hafting et al. 2005; Jeffery and Burgess 2006; Fig. 1C). The immediate impression when seeing the grid pattern for the first time is that this must be some type of grid reference, presumably for the place cell cognitive map.

The spacing between the firing fields of a given grid cell is always the same in any environment, although it tends to be smaller (~ 30 cm) for cells located more dorsally and larger (sometimes meters) in more ventral regions (Brun et al. 2008), suggesting a capacity to make maps at different scales. The importance of grid cells lies in the fact that they indicate an internal distance-measuring (odometric) process that has revealed metric space to be a genetically encoded prior in the brain. Metric space can be distinguished from topological space, in which adjacency relationships are preserved but precise distances and directions are not. From a more practical perspective, these cells provide a window into the metric structure of the map, which we will examine here. It will be shown that the map seems not to be a rigid and metrically uniform entity, like a mariners’ chart: rather, evidence is that the map is shaped and distorted by the environment. It is speculated that the way the environment exerts this influence is via the opportunities it provides, or withholds, for movement: the movement affordances.

## Boundaries

One of the most important movement constraints in an environment is the boundaries (e.g., edges of a platform or walls of a box), which confine an animal to a region and may prevent it from easily leaving: they also give the environment its geometric shape, and provide a set of fixed reference points against which to compute distance traveled. The importance of boundaries for place cells, and by implication an animal’s knowledge of its location, was first revealed in informal experiments by O’Keefe, in which he found that shifting of the platform the rat was exploring caused a concomitant shift of the place fields. This suggested that the platform, rather than the larger room it was in, defined the reference frame against which the cells were assessing where to fire. Shortly afterward, Muller and colleagues reported that place fields in a cylinder were often crescent-shaped if they were near the curved walls (Muller et al. 1987). In a subsequent more controlled “stretchy-box” experiment, O’Keefe and Burgess showed that when the walls of a box were shifted relative to each other to shrink or enlarge the box, different cells shifted their firing fields to follow different combinations of walls (O’Keefe and Burgess 1996), indicating that the cells were sensitive to wall location, and that the place cell map was deformable. Recent evidence from boundaries created by textures on the floor suggests that boundaries do not need to impede progress, but can merely indicate environmental discontinuity (Wang et al. 2020).

Grid cells also turned out to be sensitive to boundaries, as shown in the stretchy-box paradigm which revealed a partial stretching/compressing of the grids (Barry et al. 2007), albeit only in a familiar environment when the animal already knew about the relative locations of the walls. Later experiments in which rats were placed in non-rectilinear environments (such as a trapezoid) also found deformation of the grid (Jeffery 2015; Krupic et al. 2015; Stensola et al. 2015). In these cases the deformation was present right from the first exposure, suggesting an immediate tension between the boundaries and the self-motion signals. This cue combination process, in which cells combine static environmental information with dynamic self-motion signals, is a fundamental theme that runs through the spatial coding literature. A recent experiment has shown that boundaries act on grid cells by correcting errors that have crept in as the animal has explored open space (Hardcastle et al. 2015). One source of boundary information is likely to be a class of neurons reported in two brain areas near the hippocampus: subiculum and entorhinal cortex, in which some neurons fire along the walls of the environment (border cells, Solstad et al. 2008; Fig. 1C) or parallel to them at a fixed distance (boundary vector cells; Lever et al. 2009). The cells' firing is usually not along every border but only those that lie in a given direction (likely informed by another class of cells, not discussed here, called head direction cells). It may be, therefore, that grid cells and/or border cells are the means by which place cells are able to assess the distance of the animal from a boundary in a given direction.

## Environmental dimensionality

As well as boundaries and the fixed reference frame they provide, another important property of environments is their dimensionality, D. In addition to the two-dimensional (2D) environments described above, animals can also explore 1D environments (a linear track, such as the top of a wall) or 3D environments (open water or air). There are also intermediate situations in which movements in one dimension are, over time, also distributed through other dimensions. For example, the “hairpin maze” of Derdikman et al. (2006) is a series of linear tracks lying side-by-side and connected at the ends to allow the animal to cross from one to the next. Thus, most of the time the animal is running in one dimension, but each successive run briefly translates it across the second dimension. This notion can be extended into the third dimension: for example, a squirrel running along tree branches is exploring a set of 1D tracks distributed in a 3D space. Similarly, a stack of 2D surfaces one on top of the other means that a surface-running animal is, over time, moving through a volume. One more example of this intermediate dimensionality is a curved or undulating surface which at a local point in space is flat but which over time moves the animal through three dimensions. In technical terms we say that each of these environments is a lower dimensional manifold embedded in a higher dimensional space: a curved surface, for example, is a 2D manifold embedded in a 3D space. And, of course, all of these spatial dimensions are embedded in a four-dimensional space–time.

**Fig. 2:**
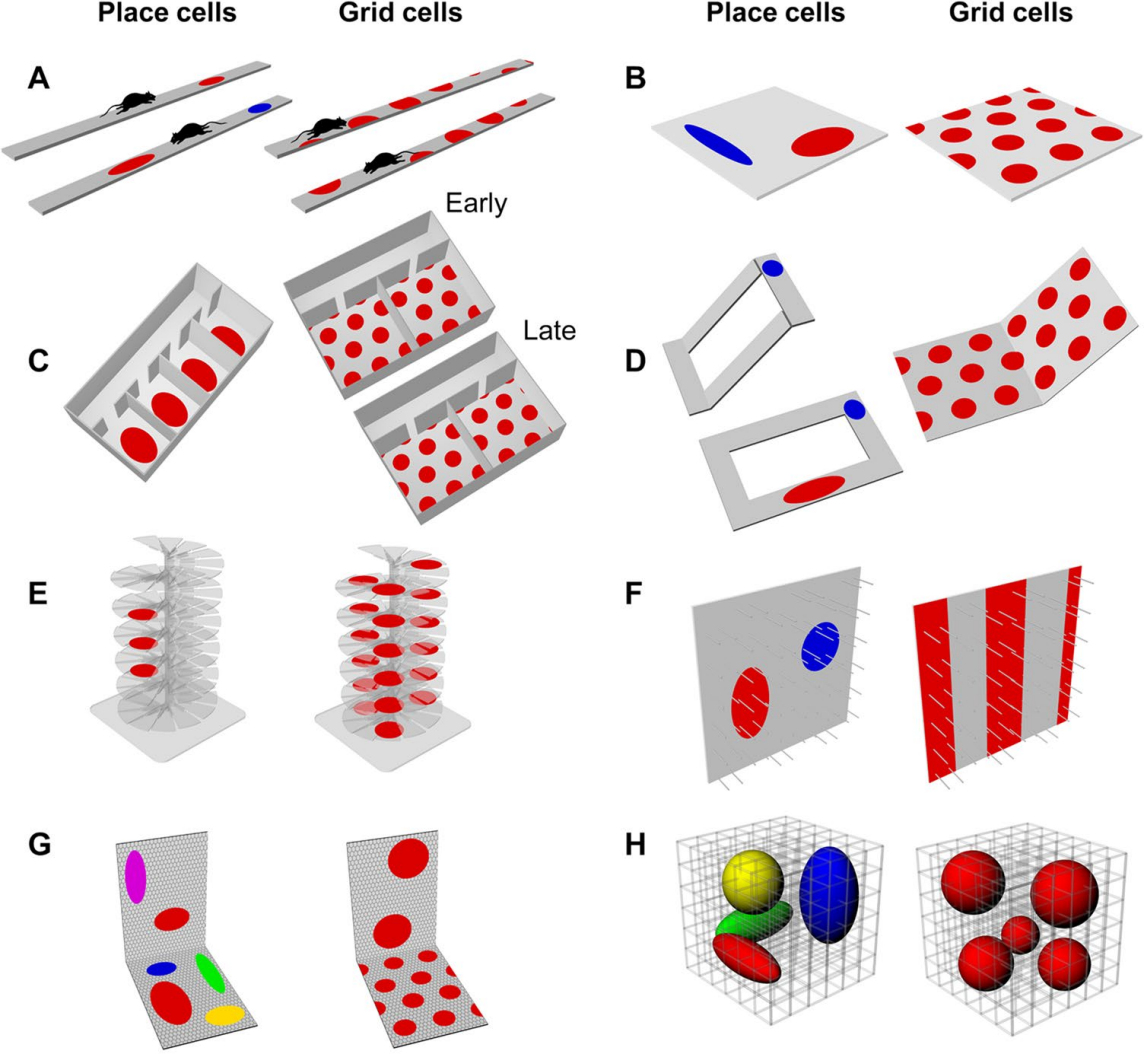
Schematic summary of experiments on place and grid cells in structured environments. **A** On a linear track, place cells tend to fire differently in one running direction than the other. Cells “remap” by either shifting their firing fields (red cell) or switching their fields on/off (blue cell). Grid cells remap by shifting their fields between running directions, but never switch on/off. **B** On a two-dimensional open arena, place cells typically (though not invariably) have singular fields, often more elongated near boundaries (Muller et al. 1987), while grid cells show the canonical hexagonal close-packed pattern (Hafting et al. 2005). **C** In a multi-compartment environment lacking any detectable distinction between compartments, place cells tend to repeat their fields in each compartment (Spiers et al. 2015; see also (Skaggs and McNaughton 1998). The same is true for grid cells early on in testing (Carpenter et al. 2015; see also Derdikman et al. 2006), but later on the grid pattern becomes continuous across the global space, and thus different in the individual compartments. **D** if a linear track is tilted, then both place cell firing fields either follow the surface (blue cell) or remap (red cell; Knierim and McNaughton 2001) while grid cells follow the surface but don’t remap (Hayman et al. 2015). **E** On a helical maze extending into the vertical dimension, place cells repeat their (usually singular) fields on a restricted number of adjacent coils, whereas grid cells produce multiple fields that repeat on every coil (Hayman et al. 2011). **F** Over a vertical surface where the body remains horizontal, place cells produce normal fields (albeit slightly vertically elongated) whereas grid cells are periodic only in the horizontal dimension, producing stripes (Hayman et al. 2011). **G** By contrast, if the animal’s body is parallel to the vertical surface then place cells produce normal-sized fields but fewer of them (reduced firing probability) and grid cells produce an expanded, probably irregular pattern (Casali et al. 2019). **H** If rats can move through a volumetric lattice then place fields are also volumetric and tend to be elongated along the “corridors” in the maze (Grieves et al. 2020) whereas grid cells form irregularly distributed blobby firing fields (Grieves et al. 2021). Source: Figshare https://doi.org/10.6084/m9.figshare.14906646 licensed under CC-BY license

An interesting question is whether the cognitive map encodes all these dimensions. The original place cell experiments were conducted in a two-dimensional (usually square or circular) space, but an early development was to reduce the environment to one dimension so as to be better able to isolate the firing properties. When recordings were made as rats traversed the arms of a radial maze it was found that many place cells showed a directionality in their firing, producing different place fields traveling in one direction than in the other (McNaughton et al. 1983; Muller et al. 1994; Fig. 2A). This was unexpected given that in two dimensions the cells fire uniformly in all traveling directions through a place field. This directionality is now thought to be a form of so-called remapping, in which the system has decided that the two traveling directions constitute two different situations. Whether the animal as a whole “thinks” these are two different environments is unknown at present, although it seems unlikely.

Grid cells on a linear track produce unevenly spaced firing fields that have statistical properties consistent with being a one-dimensional slice through a two-dimensional array of fields (Yoon et al. 2016). However, if the track is circular then the fields space themselves out evenly around it (Jacob et al. 2019), which raises the question of how the grid cell “knows” how to do this: it would to have information that extends beyond the immediate location of the rat. The answer is likely the same as the one that explains why grid cells adjust their grids if the walls of a familiar environment are moved: the system detects that there is a tension between the intrinsic propensity of the cells to keep the firing fields spaced at the correct distance and the extrinsic positioning of fields relative to features of the environment. If a conflict develops between the intrinsic signal (“it’s time to fire here”) and the extrinsic signal (“but this is too close to another firing location”) then some type of plasticity occurs to adjust the inputs to the cells so as to minimize the conflict. This type of conflict resolution is analogous to how stresses and strains are distributed in a cooling glass, and is thought to reflect a type of network behavior known as attractor dynamics (McNaughton et al. 2006; Jeffery 2011; Knierim and Zhang 2012).

What about 1D environments embedded in a 2D space? Derdikman et al. (2006) investigated what would happen to grid cells in the hairpin maze described earlier—would a 2D grid pattern emerge when the 1D patterns obtained on each track were pieced together? The answer was no: the pattern simply repeated, as if the animal’s brain had not detected the movement in the second dimension, orthogonal to the direction in which it was mainly running. Similarly, when two 2D environments were laid side-by-side, the pattern was again found to initially repeat in the two compartments (Carpenter et al. 2015; Fig. 2C), suggesting that the self-motion information from the animal running between one environment and the next was not enough to override the strong inputs from the local boundaries in each compartment. A similar effect had been seen in place cells when as many as four environments were laid side-by-side (Fig. 2C; Spiers et al. 2015). However, when the grid cell experiment was repeated over many days, the pattern slowly adapted until it eventually did become continuous, with a single grid spanning the entire two-compartment space. The implication is that the self-movement signals, which were ineffectual at first, created enough of a conflict in the network for plasticity to slowly adjust the pattern over time.

What about for a 2D environment embedded in a 3D space? The simplest situation is if a flat surface is sloped: does the system align its metric (implemented by the grid cells) to the horizontal plane or to the surface? Put another way, is the cognitive map fixed in space, regardless of how the animal is constrained to move in that space, or is it flexible and defined by the environment? The first attempt to address this question was an experiment by Knierim and McNaughton (2001) in which a horizontal environment was tilted to see if the place fields might shrink or stretch as the tilting environment sliced through progressively smaller or larger smaller sections of them, but this did not happen (Fig. 2D). Instead, cells either remapped or else they maintained their firing relative to the track surface. A similar experiment in grid cells found no change when an environment was tilted to 40°, which is extremely steep: the grid pattern did not change, and the cells seemed to follow the surface (Hayman et al. 2015). However, this did not happen when animals either climbed a helical staircase (Fig. 2E) or roamed over a vertical surface while standing on horizontal pegs, in both cases keeping the body horizontal (Hayman et al. 2011; Fig. 2F).: Unlike with place cells, which formed discrete firing fields in vertical space on both apparati, the grid cells' firing pattern instead repeated at each horizontal level, forming vertical stripes that suggested that the pattern was aligned to the floor, not the wall. It seems that, as in the Derdikman et al. hairpin maze experiment, that the system was not tracking the vertical movement; only the horizontal. However, in a different experiment in which the surface was positioned at 90° but the rats explored with their body parallel to the wall instead of the floor (by climbing on chicken-wire), now the grid pattern followed the wall. However, it was changed—the fields became larger and more widely spaced (Casali et al. 2019; Fig. 2G). Place cells, by contrast, maintained normal spatial firing patterns but were less likely to fire at all on the wall. Two findings follow from this rather complex collection of findings: first, the plane in which the grid pattern emerges seems to align with the body of the rat; and second, either the body orientation of the animal or the unusual locomotor affordances of the pegs or chicken-wire on the walls affected odometry (distance-measuring), disrupting the grid and reducing place cell activity.

Another interesting embedded 1D experiment, this time 1D embedded in 3D, was conducted on place cells during the Neurolab space shuttle mission of 1998 (Knierim et al. 2000). Here, rats ran in microgravity on a three-segment linear track in which each segment turned through 90°. The rats could cling to the Velcro surface, and the turns were positioned in three dimensions in such a way that the second turn/third leg of the track took the animal back to the starting point (on a normal flat surface, of course, three such turns are needed: e.g., left, left, and left again). The question was whether place cells would be able to stably form a map of such a 3D environment given that the system was deprived of information about gravity, and that the number of turns did not match what would normally be required. In fact, apparently normal place fields formed and persisted. It seems that the pattern was determined by the surface itself, rather than how the surface was positioned in higher dimensions.

What about if the environment is fully 3D, also known as volumetric? Examples of volumetric spaces include air and water, as well as spaces in microgravity that astronauts can float through. For land-dwelling animals, volumetric spaces can be accessed via lattice-like structures such as tree branches. Grid cells, because of their metric properties, provide a means to assess how the cognitive map is structured in 3D space, and such studies have begun to explore the 3D pattern.

Theoretical considerations suggest that grid fields might form a close-packed array in a volumetric space—the same pattern that tennis balls would form in a box, if packed in as tightly as they could go (Jeffery et al. 2013, 2015; Stella and Treves 2014; Mathis et al. 2015). There are two forms of such a pattern, both equally efficient—one is called hexagonal close-packed and the other is called face-centered cubic. In fact, evidence is emerging that the grid cell pattern may be neither of these. In bats, preliminary evidence suggests that grid cells form discrete 3D firing fields but these are distributed irregularly throughout the space. This distribution is reportedly not entirely random, as the inter-field distance is found to be more similar than would be expected from true randomness (Ginosar et al. 2017). In rats, an experiment in which the animals explored a 3D lattice found firing fields that were discrete and irregularly dispersed, but with statistics more similar to truly random (Grieves et al. 2021; Fig. 2H). These observations suggest that the grid pattern arises from self-organizing processes that are influenced by the pattern of locomotion through the space, resulting in a crystalline, semi-regular or irregular (amorphous) pattern depending on the constraints (Grieves et al. 2021). We examine this notion in more detail below.

## Movement affordances and the cognitive map

How are we to make sense of all these disparate findings? To recap (Fig. 2): on a flat surface, grid fields are flat and form a 2D hexagonal close-packed array, but if the environment is deformed or distorted then the grid also deforms, although by a lesser amount. On a sloping surface that the rat can walk on normally the grid has its normal pattern, and thus seems to have followed the surface; however on a vertical surface where the body remains horizontal (because the rat is standing on pegs) the grid fields form stripes, in which there is distance-tracking horizontally but not vertically. On a vertical surface where the body is aligned vertically (because the rat is clinging to wire on the wall) the grid is enlarged and may not be regular, and in a volumetric space the fields are globular and irregularly distributed. It thus seems that if grid cells form the metric foundation for the place cell cognitive map, then this foundation is rather malleable.

Some of the distortions of grid cell grids seem to arise from incomplete information available to the cells: for example, the stripes that formed on the pegboard (Hayman et al. 2011) or the hairpin maze (Derdikman et al. 2006) may arise from absent processing of movement in a direction orthogonal to the main locomotor direction. Relatedly, the expansion of the firing fields on the vertical wall (Casali et al. 2019) is thought to have been due to blunted speed information, caused either by the non-horizontal alignment of the head or by the altered locomotor patterns. Other distortions can be explained by reference to the interaction between self-motion information and environmental cues, when there is a conflict between these two information sources: examples are the stretching of the grid when the environment is stretched (Barry et al. 2007), the positioning of fields at equidistant spacings if the environment is circular (Jacob et al. 2019) and the deformation of the grid if the environment is not rectilinear (Jeffery 2015; Krupic et al. 2015; Stensola et al. 2015).

What about the irregularity of grid cell firing fields in a volumetric space? This was surprising, given the regular pattern they form on the floor, plus theoretical predictions indicating that an optimal organization would be the close-packed FCC or HCP patterns. However, this irregularity may be a reflection of the extra spatial degree of freedom present in three dimensions. At present, we know little about the conditions under which grid cell firing first gets established, but it is likely that intrinsic processes drive the cells to firing threshold and then extrinsic cues available at this location are attached to the cell, such that they will be able to drive it to fire here again in future. Because grid odometry evidently works best (or even perhaps only) in the direction of locomotion, it may be that the exploration path of the animal on a flat plane is sufficiently constrained to allow generation of the regular arrangement of grid fields, whereas the far more heterogeneous paths through a volumetric space are simply too irregular to allow evenly spaced firing fields to form. Similar phenomena occur in other natural physico-chemical systems. Silicon dioxide is a good example, forming (depending on the environmental conditions) crystalline quartz, locally ordered glass or amorphous silica, all from the same molecular substrate. The different patterns reflect the different constraints on the self-organization dynamics, and raise interesting questions about the mental scaling of space.

## An enactivist perspective on the cognitive map

What does all this mean for the structure of the cognitive map of space? Given the hypothesis that the grid cells provide the metric foundation—the grid reference, as it were—of the cognitive map, what are we to make of the heterogeneity of grid patterns that derive from these different types of structured environment? One thing that is certain is that the representation is not of an objectively existing, static space that is sampled when the animal moves through it. If that were the case then place and grid fields would have the same properties no matter what type of surface the animal was on or at what inclination. Rather, it seems that the map is specific to the particular circumstances of each environmental setting.

Does this specificity have functional consequences? We don’t yet know, but we can speculate that it might do, based on a principle elaborated in the 1990s known as enaction. This term was first introduced by Varela and colleagues (Varela et al. 1991) and is part of an intellectual tradition known as embodied cognition, which sees cognitive processes as arising from an interaction between the organism and its environment. Enaction, and the school of thought that derives from it, called enactivism, sits within this framework and is concerned with the active way in which sensory inputs are obtained and percepts constructed. In their words: “the overall concern of an enactive approach to perception is not to determine how some perceiver-independent world is to be recovered; it is, rather, to determine the common principles or lawful linkages between sensory and motor systems that explain how action can be perceptually guided in a perceiver-dependent world.” By this view, the organism is not a passive recipient of incoming sensory impressions that are used to construct a percept of the world “out there”—rather, it actively directs its actions in the environment, and in doing so, shapes the nature of the sensory impressions it receives. The resulting percept is thus a dialogue between the active organism and the environment it acts in.

With place and grid cells we see clear aspects of this interaction, with a role for self-generated movement in the shaping of the spatial code. For example, if an animal is moved passively by an experimenter instead of by active walking, there is degrading of the signal both for place cells (Foster et al. 1989; Terrazas et al. 2005) and grid cells (Winter et al. 2015). We also see a role for self-generated perception, in that attention to spatial cues in the environment affects spatial coding (Kentros et al. 2004). An exploring animal also chooses its route, and thus the stimuli that it is exposed to, and it may also choose its mode of locomotion (walking, flying, etc.) and thereby, again, alter the information that its senses receive. The experiments reviewed here additionally show how the structure of the environment itself shapes how the internal representation of that space is organized. The pattern of place and grid cell firing depends greatly on how the animal interacts with its environment.

Do these changes in neural coding affect the perception of the world? One would imagine so, but proving this is challenging because in animals, it requires the development of sophisticated behavioral methods to probe what the animal “thinks” and “knows.” With humans we have language, as well as other means of accessing internal perceptions such as map-drawing, but here we lack fine-grained access to the neural code (with some limited exceptions, such as patients with chronically implanted diagnostic electrodes). However the recent development of brain imaging methods to probe grid-like (“hexadirectional”) coding in human brains (Doeller et al. 2010) may enable these two lines of research to converge. Interestingly, the human studies are pointing to the possibility that the entorhinal grid code might be used for more than just physical space (Constantinescu et al. 2016), suggesting that a brain system that evolved to support movement through the real world may have become adapted, in humans, to movement through more conceptual spaces.
